# Attainment of Brown Adipocyte Features in White Adipocytes of Hormone-Sensitive Lipase Null Mice

**DOI:** 10.1371/journal.pone.0001793

**Published:** 2008-03-12

**Authors:** Kristoffer Ström, Ola Hansson, Stéphanie Lucas, Pernilla Nevsten, Céline Fernandez, Cecilia Klint, Sofia Movérare-Skrtic, Frank Sundler, Claes Ohlsson, Cecilia Holm

**Affiliations:** 1 Department of Experimental Medical Science, Lund University, Lund, Sweden; 2 National Center for High Resolution Electron Microscopy, Lund University, Lund, Sweden; 3 Research Center for Endocrinology and Metabolism, The Sahlgrenska Academy at Gothenburg University, Gothenburg, Sweden; Mayo Clinic College of Medicine, United States of America

## Abstract

**Background:**

Hormone-sensitive lipase (HSL) is expressed predominantly in adipose tissue, where it plays an important role in catecholamine-stimulated hydrolysis of stored tri- and diglycerides, thus mobilizing fatty acids. HSL exhibits broad substrate specificity and besides acylglycerides it hydrolyzes cholesteryl esters, retinyl esters and lipoidal esters. Despite its role in fatty acid mobilization, HSL null mice have been shown to be resistant to diet-induced obesity.

**Methodology/Principal Findings:**

Following a high-fat diet (HFD) regimen, energy expenditure, measured using indirect calorimetry, was increased in HSL null mice. White adipose tissue of HSL null mice was characterized by reduced mass and reduced protein expression of PPARγ, a key transcription factor in adipogenesis, and stearoyl-CoA desaturase 1, the expression of which is known to be positively correlated to the differentiation state of the adipocyte. The protein expression of uncoupling protein-1 (UCP-1), the highly specific marker of brown adipocytes, was increased 7-fold in white adipose tissue of HSL null mice compared to wildtype littermates. Transmission electron microscopy revealed an increase in the size of mitochondria of white adipocytes of HSL null mice. The mRNA expression of pRb and RIP140 was decreased in isolated white adipocytes, while the expression of UCP-1 and CPT1 was increased in HSL null mice compared to wildtype littermates. Basal oxygen consumption was increased almost 3-fold in white adipose tissue of HSL null mice and was accompanied by increased uncoupling activity.

**Conclusions:**

These data suggest that HSL is involved in the determination of white versus brown adipocytes during adipocyte differentiation The exact mechanism(s) underlying this novel role of HSL remains to be elucidated, but it seems clear that HSL is required to sustain normal expression levels of pRb and RIP140, which both promote differentiation into the white, rather than the brown, adipocyte lineage.

## Introduction

Obesity is a growing health problem globally, predisposing individuals to disorders such as type 2 diabetes, cardiovascular disease and certain cancers. Understanding the molecular events underlying adipogenesis and determination of the differentiation of white versus brown adipocytes is thus of both basic and clinical importance. Brown adipocytes are characterized by expression of UCP1 and high energy expenditure potential. Thus, attainment of brown adipocyte features in white adipocytes has evolved as a legitimate strategy to combat obesity. Considerable efforts over the last decades have led to the identification of a large plethora of transcription factors involved in differentiation and determination of adipocytes. The CCAAT/enhancer binding protein C/EBPα [1] and PPARγ [2], [3] are master regulators of adipogenesis. With regard to determination of the differentiation into the white versus the brown adipocyte lineage, key factors include transcriptional intermediary factor 2 (TIF2) [4] and the retinoblastoma protein (pRB) [5], promoting white adipocyte differentiation, and the PPARγ co-activator α (PGC-1α) [6], [7] and steroid receptor coactivator 1 (SRC-1) [4], promoting development into a brown adipocyte-like phenotype. Receptor interacting protein 140 (RIP140) antagonizes PGC coactivators and thus the appearance of a brown adipocyte phenotype [8], [9]. Very recently it was shown that brown, but not white, preadipocytes express myogenic markers, suggesting that brown and white adipocytes originate from distinct cell lineages [10].

HSL is a key enzyme in fatty acid mobilization in white adipose tissue (WAT) [11]. It has a wide tissue distribution and is, besides WAT and brown adipose tissue (BAT), expressed also in steroidogenic tissues, skeletal muscle, macrophages and pancreatic β-cells. A unique property of HSL is its broad substrate specificity, with efficient hydrolysis of a large panel of substrates, including acylglycerols, cholesteryl esters [12] and retinyl esters (RE) [13]. In recent years several strains of HSL null mice have been generated and characterized independently in several laboratories [14]–[17]. Although differences between the HSL null models exist, several common features have been reported. First, and most unexpectedly, HSL null mice are not obese. In fact, these mice have unchanged or reduced adiposity when fed a normal chow diet (ND) [14], [15], [17] and exhibit a remarkable resistance to diet-induced obesity [18] when provoked with a long-term high-fat diet (HFD) regimen. Decreased expression levels of adipogenic transcription factors and lipogenic enzymes suggested impairment of adipogenesis in HSL null mice, as also demonstrated in mice lacking both HSL and leptin [19]. A second striking feature that is shared between the existing HSL null models is male infertility due to oligospermia, demonstrating that HSL expression is necessary for normal spermatogenesis. With regard to the control of glucose homeostasis in HSL null animals conflicting data have been reported for both insulin sensitivity and insulin secretion. The strain of HSL null mice investigated in the present study exhibits mild insulin resistance that is almost fully compensated by hypersecretion of insulin [17] and no signs of impairment of glucose-stimulated insulin secretion (GSIS) [20]. Insulin resistance in HSL null mice could be a result of ectopic fat deposition, as increased amounts of intramyoceullular lipid droplets in soleus muscles [21] as well as increased amounts of triglycerides in liver [17], [18], have been observed in HSL null mice. However, in contrast to the observations in our strain of HSL null mice, increased insulin sensitivity has been reported for two other strains of HSL null mice [22], [23] and Roduit et al. reported elevated basal insulin secretion and blunted GSIS [24]. The reason for these discrepancies remains to be resolved, but differences in nutritional state, gender, age, experimental setup, genetic background and intestinal microflora of the animals are likely to be contributing factors.

Here we extend the phenotypic characterization of HSL null mice and demonstrate that WAT of HSL null mice attains BAT characteristics. The expression levels of markers that are characteristic of brown adipocytes are elevated, whereas markers of white adipocytes are decreased. Functionally, WAT of HSL null mice exhibits increased oxygen consumption and increased uncoupling activity. Although the underlying mechanisms remain to be resolved, these data identify HSL as a determinant in the decision of cell fate during adipocyte differentiation.

## Results

### HSL null mice are resistant to diet-induced obesity

**Figure 1:**
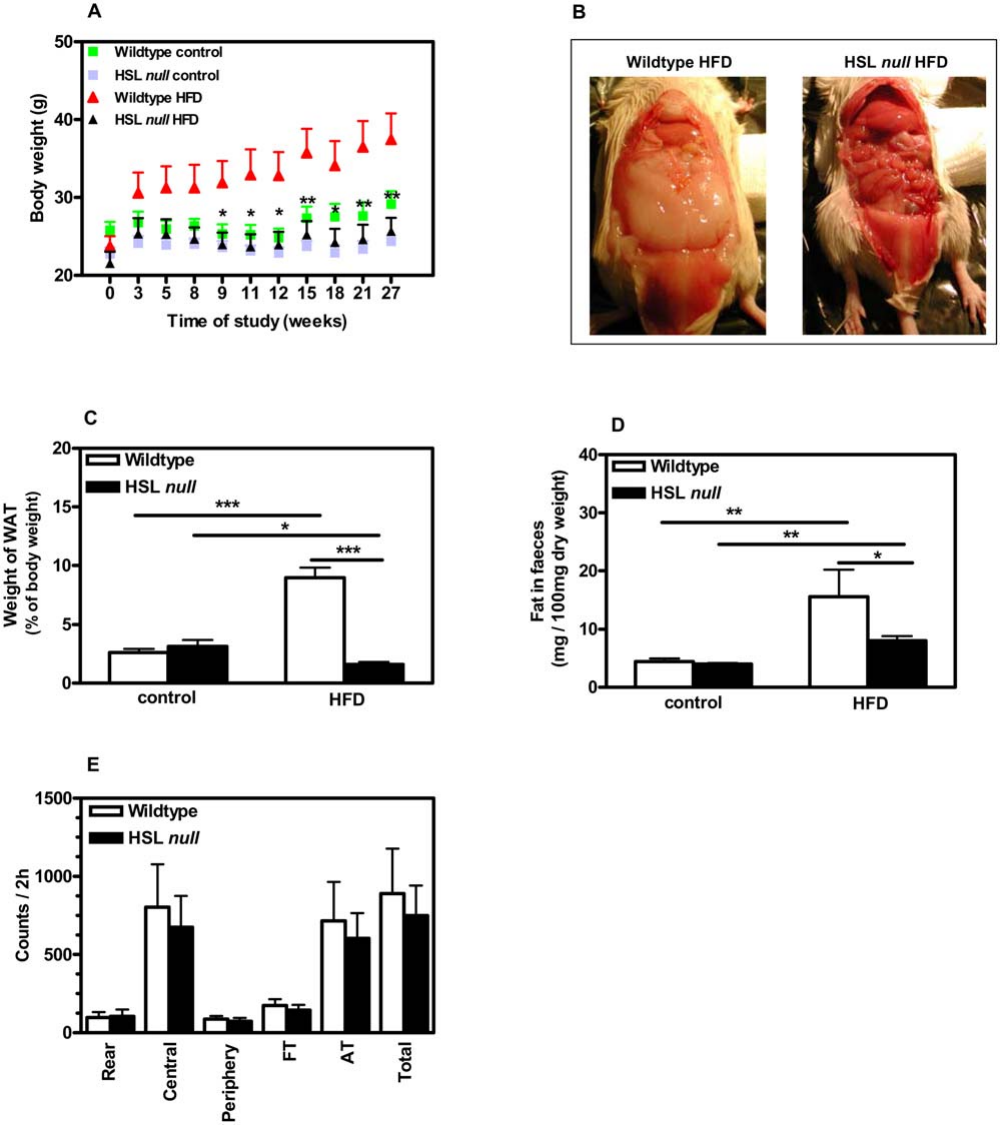
HSL null mice are resistant to diet-induced obesity. (A) Body weights of female wildtype mice (green) and HSL null mice (blue) fed control diet and wildtype mice (red) and HSL null mice (black) fed HFD measured during 27 weeks (n = 7). Significance between body weight of wildtype and HSL null mice fed HFD is indicated. (B) Female wildtype and HSL null mice at 9 months of age after 24 weeks on HFD. (C) Weight of periovarial WAT from 10–11 months old mice fed a HFD for 6 months (n = 13–17). (D) Lipid analysis on stools from 7–8 months old female wildtype and HSL null mice fed either a control diet or a HFD for 4 months, collected during a 24 h period (n = 5). (E) Registration of physical activity in 10–11 months old male mice fed a HFD for 6 months (n = 3). The parameters investigated were: horizontal motor activity in the periphery (Periphery) and in the central part (Central) of the cage, rear leg (Rear), fine (grooming) (FT), ambulatory (AT) and total (Total) movements. Values given are mean±SEM, * p<0.05, ** p<0.01, *** p<0.001, analyzed with One way ANOVA followed by Bonferroni's Multiple Comparison Test (A) and Mann-Whitney U tests (C,D,E).

**Figure 2:**
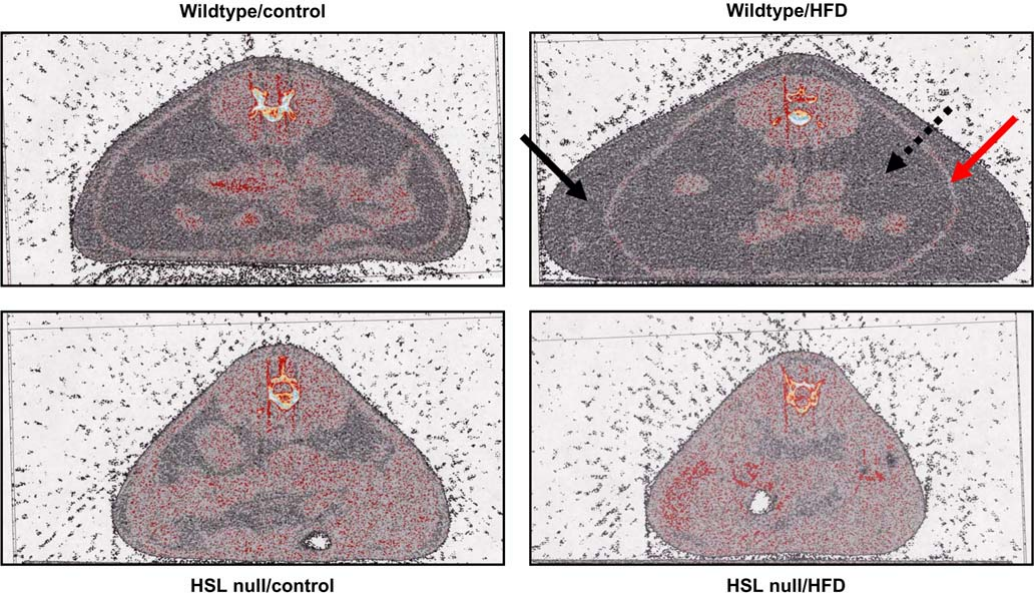
Subcutaneous and visceral adipose tissue stores are decreased in HSL null mice. Representative CT scan images displaying body fat distribution in 10–11 months old female wildtype and HSL null mice fed either a control diet or a HFD for 6 months (n = 7). The filled black arrow and the dotted black arrow indicate the location of the subcutaneous and visceral WAT, respectively. The red arrow indicates the location of the muscle layer separating the two adipose tissue depots.

As already reported in an independently generated strain of HSL null mice [18], our strain of HSL null mice displayed a significant reduction in both body weight (**Figure 1A**) and WAT mass (**Figure 1B,C**) compared to wildtype mice following a 6-month HFD regimen. This was not due to decreased food intake (data not shown), impaired intestinal lipid absorption (**Figure 1D**) or increased physical activity (**Figure 1E** and [21]) as has been shown also for another HSL null mouse model [18]. In fact, after 5 months of HFD feeding, the caloric intake per total body weight was increased by 40% in HSL null mice compared to wildtype littermates (data not shown). Also, the lipid content in faeces from HFD-fed HSL null mice was significantly lower than that of wildtype littermates (**Figure 1D**). Computer tomography (CT) scanning showed that the reduction in WAT mass in HSL null mice was more dramatic for subcutaneous depots, which were virtually absent following HFD feeding, than for visceral depots (**Figure 2**).

**Figure 3:**
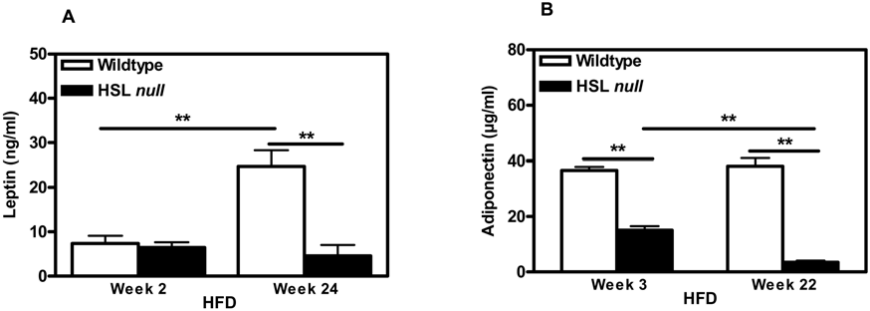
Plasma levels of adipokines are altered in HSL null mice. (A) Plasma leptin protein levels in the fed state after 2 and 24 weeks of HFD feeding in female wildtype and HSL null mice, fed the HFD from 16 weeks of age (n = 6). (B) Plasma adiponectin protein levels in the fed state after 3 and 22 weeks of HFD feeding in female wildtype and HSL null mice, fed the HFD from 16 weeks of age (n = 6). Values given are mean±SEM, ** p<0.01, analyzed with Mann-Whitney U tests.

Plasma leptin levels in the mice on control diet were similar for the two genotypes (data not shown). After 24 weeks on HFD, wildtype mice showed an expected large increase in leptin levels, whereas this increase was absent in the HSL null mice (**Figure 3A**). The plasma adiponectin level was dramatically lowered (0.41, p<0.01) already after 3 weeks of HFD feeding, decreasing even more (0.09, p<0.01) after 22 weeks of HFD, in the HSL null mice compared to wildtype littermates (**Figure 3B**). This decrease was also observed in HSL null mice fed a control diet (data not shown), although the effect was more pronounced following HFD feeding.

### Decreased expression of adipogenic markers in WAT of HFD-fed HSL null mice

**Figure 4:**
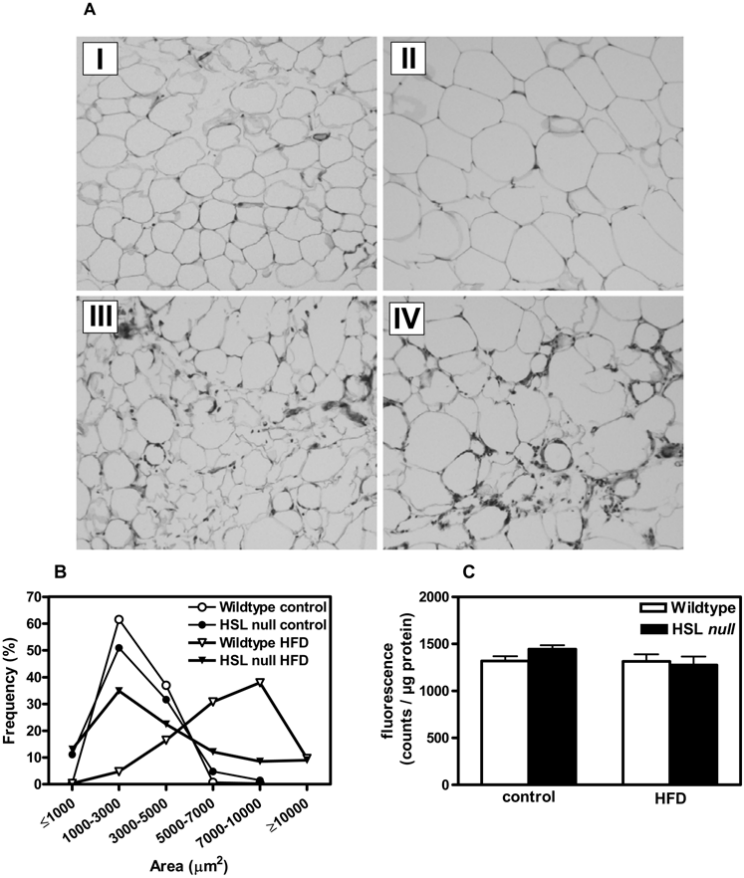
Morphological changes in WAT of HFD-fed HSL null mice. (A) Representative morphology images of epididymal WAT from 10–11 months old wildtype (I) and HSL null (III) mice fed control diet and wildtype (II) and HSL null (IV) mice fed HFD for 6 months. (B) The cell area was determined using morphometry and was based on a total of 268 cells in wildtype control, 477 cells in HSL null control, 922 cells in wildtype HFD and 775 cells in HSL null HFD. (C) Caspase 3/7 activity was investigated in the infranatant fraction of WAT from 10–12 months old female mice fed a control diet or a HFD for 6 months (n = 7–12). Values given are mean±SEM.

Using light microscopy the size of periovarial WAT adipocytes was estimated (**Figure 4A**). In the control diet groups, the median adipocyte size, measured as cell area, was similar for the two genotypes (2774±48 and 2568±68 µm^2^ for wildtype and HSL null, respectively), and although an increased heterogeneity in cell size could be seen in adipocytes from HSL null mice (**Figure 4A** **, panel I and III)**, the size distribution pattern for the two genotypes was similar with a large peak in frequency seen at 1000–3000 µm^2 ^(**Figure 4B**). After HFD feeding, adipocytes from wildtype WAT showed the characteristic large and general increase in size (**Figure 4A** **, panel II**), reflected in a shift of the curve to the right, with the highest frequency at 7000–10000 µm^2 ^(median area 6880±86 µm^2^) (**Figure 4B**). This was not observed to the same extent in adipocytes from HSL null mice, which instead exhibited an even greater size heterogeneity than that observed on the normal diet, with a markedly reduced median cell area compared to wildtype mice (3143±127 µm^2^) (**Figure 4A** **, panel IV**) (**Figure 4B**).

In WAT of HSL null mice, HFD feeding also promoted the appearance of an increased number of eosinophilic-like cells, reflecting an inflammatory state of the tissue, as previously shown [25], [26]. The inflammatory state was accompanied by macrophage infiltration and syncytia formation, supporting a necrotic-like cell death as also observed by immunohistochemistry [25]. This is further confirmed in the present study, showing no difference in caspase 3/7 activity between the two genotypes in either feeding category (**Figure 4C**).

**Figure 5:**
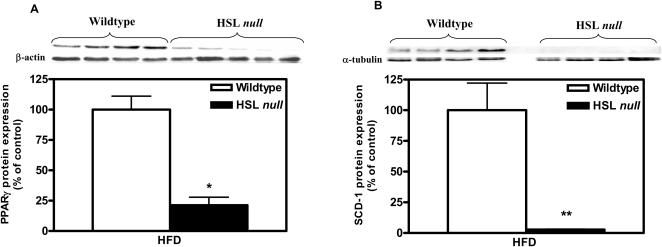
Impairment of adipogenesis in WAT of HFD-fed HSL null mice. Protein levels of PPARγ (A) and SCD-1 (B), analyzed with western blot in WAT from 10–12 months old female, wildtype and HSL null mice fed HFD for 6 months (n = 4–6). Values given are mean±SEM, * p<0.05, ** p<0.01, analyzed with Mann-Whitney U tests.

As already shown in independently generated HSL null mouse strains [18], [27], the mRNA expression levels of PPARγ and C/EBPα were several fold decreased in WAT from HFD-fed HSL null male and female mice compared to wildtype littermates (data not shown). The downregulation of PPARγ expression was confirmed at the protein level (0.21, p<0.05) using western blot analysis (**Figure 5A**). Stearoyl CoA Desaturase 1 (SCD-1), the expression of which is known to be positively correlated to the differentiation state of the adipocyte [28], was severely downregulated at the protein level (0.027, p<0.01) in WAT from HSL null mice (**Figure 5B**).

### HFD-fed HSL null mice have increased energy expenditure and increased oxygen consumption in WAT

**Figure 6:**
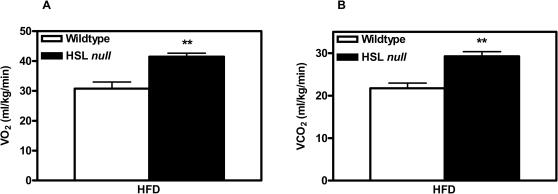
HFD-fed HSL null mice have increased energy expenditure. Indirect calorimetry measurements of 10 months old wildtype and HSL null male mice fed HFD for 6 months. Whole body oxygen consumption (A) and carbon dioxide production (B) measured for 2 h. Data from the last hour is presented (n = 7). Values given are mean±SEM, ** p<0.01, analyzed with Mann-Whitney U tests.

Indirect calorimetry measurements revealed a significant increase in O_2_ consumption (**Figure 6A**) and CO_2_ production (**Figure 6B**) in HFD-fed HSL null mice compared to wildtype littermates. No difference was seen between animals fed the control diet (data not shown).

**Figure 7:**
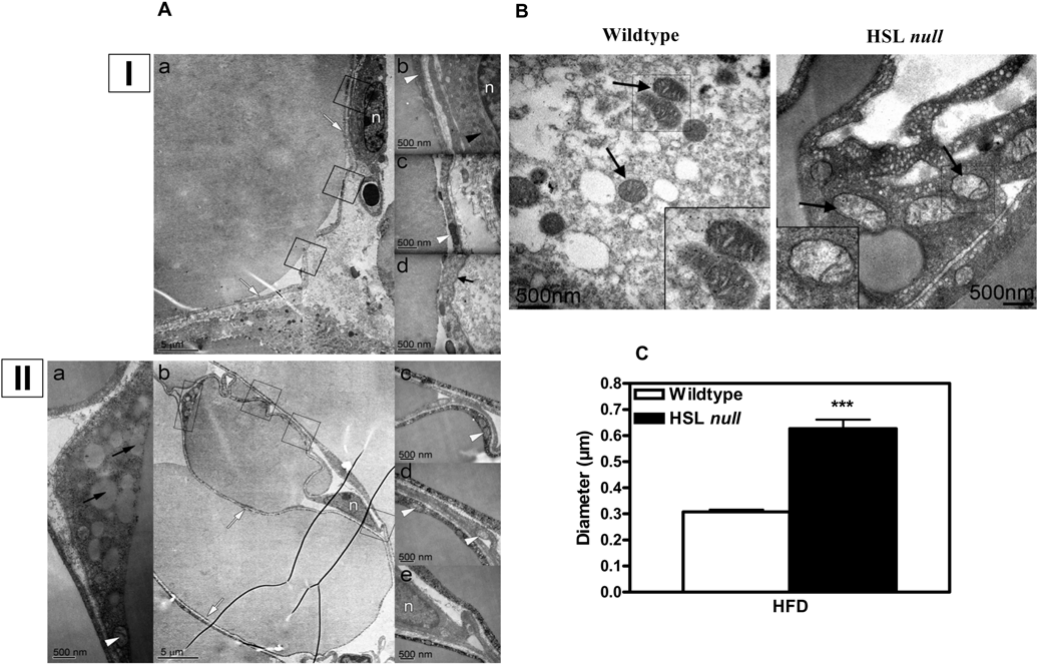
Increased mitochondrial size in adipocytes from WAT of HFD-fed HSL null mice. (A) Representative electron microscope (TEM) images of periovarial WAT from 10–12 months old wildtype (I) and HSL null mice (II) fed HFD for 6 months. (I)a shows a low-magnification overview image with details from the boxed-in areas shown in (I)b, c and d. For the HSL null mice, (II)b is the overview image and (II)a, c, d and e are the details from the boxed-in areas. Black arrows indicate small lipid droplets in the cytoplasm, black short arrow indicates caveolae, black arrowhead indicates ER, white arrows indicate cytoplasm, white arrowheads indicate mitochondria and n indicates the nucleus. (B) Representative TEM image of periovarial WAT from 10–12 months old wildtype and HSL null mice fed HFD for 6 months. Black arrows indicate location of mitochondria. Insets show enlargement of representative mitochondria. (C) The diameter of mitochondria in WAT was determined using morphometry and based on 140 wildtype and 105 HSL null mitochondria. Values given are mean±SEM, *** p<0.001, analyzed with Mann-Whitney U tests and multiple regression analysis.

Periovarial WAT from female HFD-fed mice was collected and analyzed by transmission electron microscopy (TEM) (**Figure 7A**). An increase in the size of the mitochondria from HSL null mice compared to wildtype littermates was observed (**Figure 7B**), and estimated to 50% (p<0.001) using morphometric analysis (**Figure 7C**). Cristae, however, appeared less dense in mitochondria from HSL null mice (**Figure 7B**).

**Figure 8:**
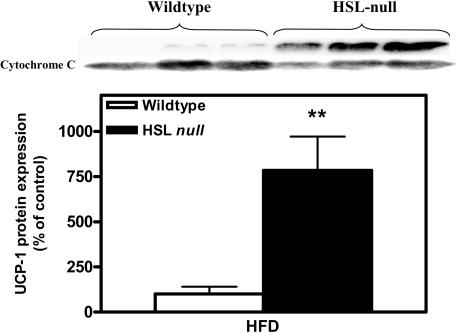
Induction of UCP-1 in WAT of HSL null mice. Levels of UCP-1 protein in the mitochondrial fraction analyzed with western blot in periovarial WAT from 9–10 months old, wildtype and HSL null mice fed HFD for 6 months (n = 5). Values given are mean±SEM, ** p<0.01, analyzed with Mann-Whitney U tests.

**Figure 9:**
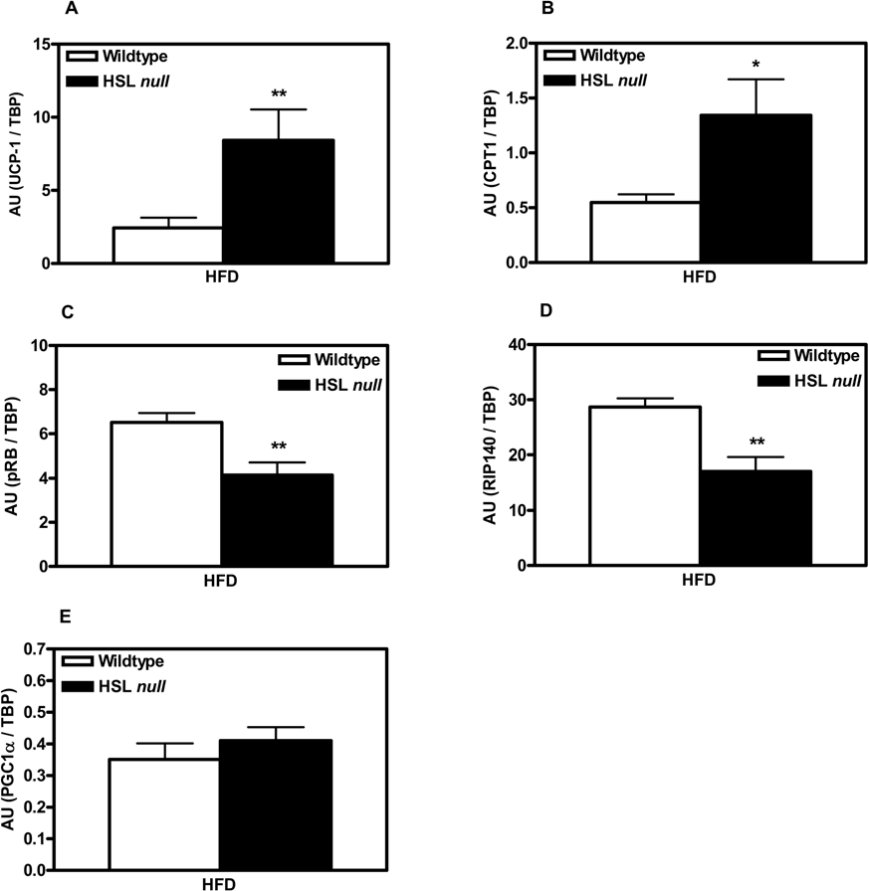
Altered gene expression in white adipocytes from HSL null mice. mRNA level of UCP-1 (A), CPT1 (B), pRb (C), RIP140 (D) and PGC1α (E) analyzed with real-time quantitative PCR in isolated adipocytes from periovarial WAT from 2 months old, wildtype and HSL null mice fed HFD for 4 weeks (n = 6). Values given are mean±SEM, * p<0.05, ** p<0.01, analyzed with Mann-Whitney U tests.

The change in color of WAT towards a more brownish appearance together with the increase in size of mitochondria from HFD-fed HSL null mice, led us to hypothesize that WAT of HSL null mice had attained BAT characteristics. To investigate this, we first analyzed the protein expression of UCP-1, the highly specific marker of BAT, in WAT using western blot analysis. UCP-1 protein expression was found to be more than 7-fold increased in HFD-fed HSL null mice compared to wildtype littermates (**Figure 8**). Next we analyzed the mRNA expression of UCP-1 and other proteins known to be differentially expressed between white and brown adipocytes using real-time quantitative PCR. To confidently monitor the mRNA expression in adipocytes, without contribution from other cell types present in adipose tissue, these analyses were performed on isolated adipocytes obtained from animals that had been on HFD for 4 weeks. At this early time point in the study it is possible to obtain a representative population of adipocytes following collagenase treatment of adipose tissue, whereas this is difficult following a 6-month HFD regimen due to the presence of very large adipocytes that are more sensitive to collagenase treatment. As shown in **Figure 9A**, the UCP-1 mRNA levels were found to be increased 3.5-fold (p<0.01) in white adipocytes of HSL null mice compared to wildtype mice. Also, carnitine palmitoyl transferase 1 (CPT1), the rate-limiting enzyme for long-chain acyl-CoA transport into mitochondria, known to be more highly expressed in brown than in white adipocytes [29], showed a higher expression in HSL null than in wildtype white adipocytes (2.2-fold, p<0.05) (**Figure 9B**). A functional reflection of this could be the increased oxidation of palmitate observed in epididymal and periovarial isolated white adipocytes of 6 months old HSL null mice fed a normal chow diet (28.210±4.616 pmol CO_2_/h/µl packed cell volume (PCV), n = 3) compared to wildtype littermates (9.443±3.260 pmol CO_2_/h/µl PCV, n = 3, p<0.05), in the fed state. The mRNA levels of pRb, shown to act as a molecular switch determining white versus brown adipocyte differentiation [5], and RIP140, which also promotes differentiation into the white adipocyte lineage [9], were decreased by 36 and 40% (p<0.01), respectively, in white adipocytes of HSL null mice compared to wildtype littermates (**Figure 9C,D**). The expression of PGC-1α, on the other hand, was not significantly different between the two genotypes (1.17, p = 0.36) (**Figure 9E**).

**Figure 10:**
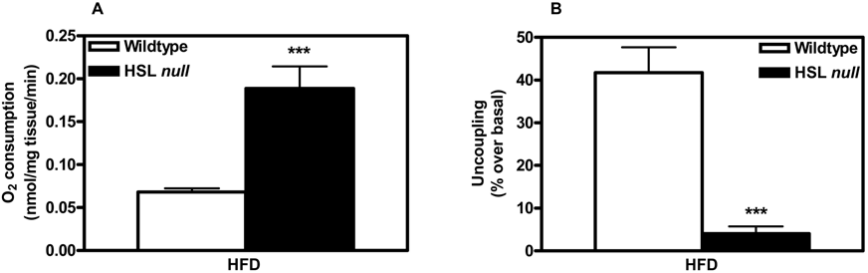
HFD-fed HSL null mice have increased oxygen consumption in WAT. Oxygen consumption (A) and level of uncoupling of mitochondria (B) measured using a Clark electrode in periovarial WAT pieces from 10–11 months old, wildtype (n = 11 and 9 respectively) and HSL null mice (n = 11 and 8 respectively) fed HFD for 6 months. Values given are mean±SEM, *** p<0.001, analyzed with Mann-Whitney U tests.

In order to study the functional consequences of the increased UCP-1 expression, oxygen consumption was investigated on isolated WAT from HFD-fed mice. Basal oxygen consumption was almost 3-fold increased (p<0.001) in WAT from HSL null mice compared to wildtype littermates (**Figure 10A**). The level of electron transport chain uncoupling in WAT from the two genotypes was investigated by adding the chemical uncoupler FCCP. Addition of FCCP increased the oxygen consumption by almost 50% in WAT from HFD-fed wildtype mice, whereas the same uncoupler had almost no effect in WAT from HFD-fed HSL null mice (**Figure 10B**).

Taken together, these data suggest that WAT of HSL null mice fed a HFD attains BAT characteristics that are functionally manifested as increased oxygen consumption and increased uncoupling activity.

## Discussion

Resistance to diet-induced obesity, accompanied by impaired adipogenesis, has been reported previously in two independently generated lines of HSL null mice [18], [19], [30] and confirmed here in our line of HSL null mice. Furthermore, we have used indirect calorimetry to demonstrate that HFD-fed HSL null mice indeed exhibit increased energy expenditure, as previously suggested by the observed fasting-induced weight loss and increased body temperature [18].

A novel and key finding of our paper is the demonstration of attainment of BAT characteristics of WAT of HSL null mice. There are several manifestations of acquisition of BAT characteristics in WAT of HSL null mice. First, already macroscopically WAT of HSL null mice has a more brownish appearance than WAT from wildtype littermates. Second, the expression of pRb and RIP140, which directly and indirectly, respectively, promote differentiation into the white versus the brown adipocyte lineage (for a review see [8], [31]–[33]), is downregulated in white adipocytes from HSL null mice. Third, the expression of UCP-1 and CPT1 is increased in adipocytes from classical WAT depots of HSL null mice compared to wildtype littermates. Fourth, and most importantly, oxygen consumption and uncoupling activity are increased in WAT of HSL null mice. In BAT, on the other hand, there were no signs of increased oxygen consumption in HFD-fed HSL null mice (data not shown). The increased size of mitochondria of white adipocytes may be an additional manifestation of attainment of brown adipocyte features in white adipocytes, although cristae appeared less dense in null cells.

Several different mechanisms may underlie the decreased adiposity of HSL null mice, including the attainment of BAT characteristics of WAT demonstrated in this study. Another mechanism suggested to contribute to decreased fat stores of HSL null mice is the dramatic reduction in reesterification of fatty acids in WAT, which in turn is due to the coordinated alteration in the expression of enzymes involved in lipogenesis [27]. It was suggested that the downregulation in reesterification was compensatory to the reduced lipolytic activity, thus serving to uphold the plasma concentrations of fatty acids even in the absence of HSL. Also the upregulation of lipoprotein lipase observed in the fasted state in WAT of HSL null mice, may be compensatory to the lack of HSL activity. However, ATGL, which together with HSL has been shown to account for 95% of triglyceride lipase activity of murine WAT [34], appears to be unchanged in WAT of HSL null mice [35]. Yet another mechanism that may contribute to decreased adiposity of HSL null mice is the described adipocyte cell death in WAT of HSL null mice [25], which appears to be necrosis rather than apoptosis [25] (**Figure 4C**). Emergence of brown adipocytes in WAT depots has been associated to a lean phenotype in several transgenic mouse models [4], [36]–[38]. Even so, the increased oxygen consumption in WAT of HSL is likely to contribute little to the increase in total energy expenditure, considering the small contribution of WAT to whole body energy expenditure compared to that of skeletal muscle, which accounts for the majority of energy consumption at the whole body level. The relative contribution of each organ to the increased energy expenditure in HSL null mice will require the generation of mouse lines in which the HSL gene has been inactivated tissue-specifically.

Altogether the phenotype of the HSL null mice is very reminiscent of that of RIP140 null mice, which also exhibit acquirement of BAT characteristics in WAT without increased PGC-1α expression [9]. Another similarity between HSL null mice and RIP140 null mice is that subcutaneous WAT depots are more reduced than visceral depots. RIP140 is expressed in both brown and white adipocytes [9] and seems to antagonize the function of PGC1 coactivators in these cells, thereby suppressing UCP-1 expression and ATP uncoupling [8]. It has been observed that in the absence of RIP140, altered expression of the PGC1 coactivators is not essential for UCP1 expression [39]. Thus, it can be speculated that downregulation of RIP140 in the HSL null mouse model, allows the low levels of PGC1 coactivators in white adipocytes to promote the appearance of brown fat features. The mechanism whereby HSL is involved in the determination of cell fate during adipocyte differentiation remains to be explored. The fact that both pRb and RIP140 are transcriptionally controlled by retinoic acid [5], [40], [41], together with our recent observation that retinyl ester hydrolase activity is dramatically reduced in WAT of HSL null mice [26], makes future studies into the role of the retinyl ester hydrolase activity of HSL highly warranted. Nevertheless, the key finding of the present paper, i.e. the attainment of BAT characteristics in WAT of HSL null mice, emphasizes that the traditional view of HSL, as a fatty acid-mobilizing enzyme in WAT, has to be abandoned in favor of an emerging view of a multifunctional enzyme with several functions within and outside WAT.

## Materials and Methods

### Animal experiments

The study was reviewed and approved by the Ethical Committees in Malmö/Lund, Lund, Sweden (license no. M162-05) and Gothenburg, Gothenburg, Sweden (license no. 294-2003) and is in accordance with the Council of Europe Convention (ETS 123). HSL null mice were generated by targeted disruption of the HSL gene in SV129-derived embryonic stem cells as described elsewhere [42]. Animals used were all from the same embryonic stem cell colony and animals in the different groups were littermates and had a mixed genetic background from the inbred strains C57BL/6J and SV129 [17]. The animals were maintained in a temperature-controlled room (22°C) on a 12-h light-dark cycle. Mice, 16–20 weeks of age, were fed *ad libitum* a chow diet (control) (11% energy from fat) or a high fat diet (HFD) (58% energy from fat) (Research Diets; products no D12310 and D12309 respectively), for 6–10 months before the *in vitro* analyses were performed. Body weights were measured every third week during the study. A shorter HFD study was also performed, where four week old female mice were fed *ad libitum* a HFD (see above) for 4 weeks before the *in vitro* analyses were performed. From these mice, RNA was extracted from isolated periovarial white adipocytes and gene expression was analyzed by real-time quantitative PCR.

For the comparison of food intake, female mice of the same genotype and feeding category were housed together and food consumption was measured as weight of the distributed food subtracted by weight of the remaining food over one week. Food consumption is presented as caloric intake per week. For measurements of stool lipids, female mice were put in metabolic cages and faeces were collected during a 24 h period. Total stool lipids were extracted and quantified according to Schwarz et al [43]. Registration of physical activity was measured during the dark-cycle by number of photo-beam disruptions using an open field (0.4 m×0.4 m) 16×16 photo-beam system (San Diego Instruments) and was performed using male mice housed individually in cages. The animals were allowed an adaptation period of 1 h before the recording started. Movements were then recorded for a period of 2 h with 4 individuals of each genotype. The parameters investigated were: horizontal motor activity in the periphery and in the central part of the cage, rear leg, fine (grooming), ambulatory and total movements.

Collected tissues were rapidly dissected, snap frozen and stored in liquid nitrogen before analyses. Blood samples were drawn by retro-orbital puncture from female animals in the fed state and plasma leptin and adiponectin concentrations were measured using a leptin ELISA kit (Crystal Chem Inc.) and an adiponectin RIA kit (Linco). To analyze body fat distribution, computer tomography (CT) was performed in female mice, 4-mm proximal to the iliac crest, identified by a longitudinal prescan, with the Stratec peripheral quantitative CT (Norland Medical Systems) at 70-µm resolution. Images were analyzed using software from Bergström Instruments (Solna, Sweden). For measurement of energy expenditure, male wildtype and HSL null mice, fed *ad libitum* the HFD, were put in metabolic cages, where oxygen consumption (VO_2_) (ml/kg/min) and carbon dioxide production (VCO_2_) (ml/kg/min) were measured for 2 h by an indirect calorimetry method (Oxymax, Columbus Instrument). To minimize influence of animal handling on the data, results from the last hour are presented.

### Morphology and morphometry of WAT

Epididymal WAT was fixed in 4% buffered formaldehyde, pH 7.2, overnight, then dehydrated in graded ethanols and embedded in paraffin. Sections (10 µm) were cut and mounted on slides, after which they were stained with hematoxylin and eosin. Slides were analyzed using an Olympus BX60 microscope and images were captured with a DS-2Mv camera (Nikon). Calculation of the area of the adipocytes was performed using NIS-Elements Advanced Research software (Nikon) (10×magnification).

### Transmission Electron Microscopy

Periovarial WAT from HFD-fed wildtype and HSL null mice were processed in parallel according to the following scheme. Following fixation in PBS containing 3% glutaraldehyde for 1 h, the tissues were cut into smaller pieces and then further fixed over night at 4°C. Specimens were post-fixed with osmium tetraoxide (1% in PBS-buffer) for 2 h at room temperature, followed by washing three times with MilliQ-water for 1 h. Dehydration was performed in acidified 2.2-dimethoxypropane (DMP) for 30 min at RT, followed by rinsing with acetone twice for 15 min. Spurr's resin was infiltrated gradually and then cured at 70°C for 16 h. Sections (1 µm) were cut and studied in a light microscope (Leica ATC 2000) to locate well-fixed tissue in the blocks, for further trimming with a glass knife. Thin sections (70 nm) were cut with a diamond knife at an ultramicrotome (Leica UCT) and collected on pioloform-filmed copper grids. The sections were then observed in a Philips CM 120 Biotwin instrument at 120 kV. Morphometric analysis was performed semi-automatically in DigitalMicrograph (GATAN), by outlining profiles to obtain data about mitochondrial diameter.

### Tissue homogenization preparation

Tissues were homogenized in 0.25 M sucrose, 1 mM EDTA pH 7.0, 1 mM dithiothreitol, 20 µg/ml leupeptin, 10 µg/ml antipain, 1 µg/ml pepstatin A using a glass/teflon homogenizer (10–20 strokes), followed by a centrifugation at 10 000 g for 25 min at 4°C. The infranatant fraction was used for caspase 3/7 activity assays and western blot analysis (SCD-1). The pellet fraction was solubilized in homogenization buffer containing 5% SDS for 30 min at 50°C, and was used for western blot analysis (PPARγ). Preparation of fractions enriched in mitochondria, was performed according to Pecqueur et al [44]. Protein determination was performed using the BCA assay (Pierce).

### Caspase-3/7 activity assay

Caspase-3/7 activity was measured using Caspase-3/7 Assay (Promega Corp) according to the manufacturer's protocol. Infranatant fractions from periovarial WAT homogenates (50 µg protein/well) were transferred to a black 96-well plate (FLUOTRAC 600, Nunc) for detection of caspase 3/7-activity. Fluorescence was measured using a fluorescence microtiter plate reader (Victor 2, Wallac, Perkin-Elmer).

### Western blot analysis

Periovarial WAT was homogenized and 25–50 µg of total protein were resolved by SDS-PAGE and electroblotted onto nitrocellulose membranes (HyBond-c extra, Amersham Pharmacia Biotech). Primary antibodies used were peroxisome proliferator activated receptor gamma (PPARγ) (1∶200), stearoyl-CoA desaturase-1 (SCD-1) (1∶200), cytochrome-c (Cyt-C) (1∶1000) (Santa Cruz Biotechnology), UCP-1 (1∶2500) (a kind gift from Prof. Barbara Cannon, Stockholm University, Stockholm, Sweden), β-Actin (1∶5000) and α-tubulin (1∶2000) (Sigma). Actin, tubulin and cytochrome-C were used as loading controls. Secondary antibodies were horseradish peroxidase-conjugated anti-rabbit IgG (Cyt-C, PPARγ and UCP-1), anti-mouse IgG (α-tubulin and β-Actin) (Amersham) or anti-goat IgG (SCD-1) (Santa Cruz Biotechnology). Western blot analysis was performed using a chemiluminescence system (Luminol) and detection was made using a CCD-camera (LAS 1000, Fuji). Relative protein levels were calculated after normalization to the loading control.

### Oxygen consumption and uncoupling in WAT pieces

Periovarial WAT was collected from female wildtype and HSL null mice from both feeding categories. Pieces of tissue (∼20–30 mg each) were incubated in Krebs-Ringer bicarbonate buffer pH 7.4 (KRH, 25 mM hepes, 10 mM glucose, 10 mM fructose, 25 mM NaHCO_3_ and 4% fatty acid free BSA (Roche)) [45]. Immediately before each assay the tissue was further cut (25 times). O_2_ consumption was measured polarographically by an electrode (Oxygraph, Hansatech instruments) in an air-sealed chamber containing 400 µl of buffer, under agitation. The calibration of the electrode was performed as described in [46]. After recording of the basal respiration rate, uncoupled respiration was measured by the addition of a chemical uncoupler FCCP (Carbonyl cyanide p-trifluoromethoxyphenylhydrazone, Sigma), to a final concentration of 50 µM. Measurement was stopped after 36 min. As a measure of repeatability, the average value of duplicate measurements of the same fat pad, from a total of 10 individuals, gave a value of 1±0.18 nmol/mg tissue/min. The reproducibility, calculated from the SD values, was 1±0.21 nmol/mg tissue/min (n = 11) for wildtype samples and 1±0.46 nmol/mg tissue/min (n = 11) for HSL null samples.

### RNA preparation and Real-time quantitative PCR (rtPCR)

Total RNA was isolated using RNeasy Lipid Tissue Mini Kit (Qiagen) according to the manufacturer's recommendations. Total RNA (1 µg) was treated with DNase I (DNase I amplification grade, Invitrogen) and then reversely transcribed using random hexamers (Amersham Biosciences) and SuperScript™II RNaseH reverse transcriptase (Invitrogen Life Technologies) according to the manufacturer's recommendations. rtPCR was performed using an ABI 7900 system (Applied Biosystems), using either Taqman probe and primers (pRb, RIP140, PGC-1α and TBP, assays-on-demand, Applied Biosystems) or SYBR green reagent (PPARγ, C/EBPα, UCP-1 and CPT1). Primer sequences, designed using the software Primer Express 1.5 (Applied Biosystems), are listed below. Relative abundance of mRNA was calculated after normalization to TATA box binding protein (TBP).

### Primers used in real-time quantitative PCR experiments

List of assays using Taqman probes (Applied Biosystems):
**pRb** (Assays-on-demand, Mm00485586_m1)
**RIP140** (Assays-on-demand, Mm00476537_s1)
**PGC-1α** (Assays-on-demand, Mm00447183_m1)
**TBP** (Assays-on-demand, Mm00446973_m1)

List of primers for gene expression analysis using SYBR green reagent:

**PPARγ**: (forward primer: 5′-CTG TTT TAT GCT GTT ATG GGT GAA A-3′ and reverse primer: 5′-GCA CCA TGC TCT GGG TCA A-3′
**C/EBPα**: (forward primer: 5′-ATA GAC ATC AGC GCC TAC ATC GA-3′ and reverse primer: 5′-CTG TCG GCT GTG CTG GAA-3′)
**mUCP-1**: (forward primer: 5′-CCT GCC TCT CTC GGA-3′ and reverse primer: 5′-TGT AGG CTG CCC AAT-3′)
**CPT1**: (forward primer: 5′-CCA AAA CAG TAT CCC-3′ and reverse primer: 5′-AAG AGA CCC CGT AGC-3′)

### Preparation of isolated white adipocytes

Adipocytes from periovarial WAT were isolated after incubation in Krebs-Ringers solution (pH 7.4) supplemented with 3.5% BSA, 2 mM glucose, 200 nM adenosine and collagenase (1 mg/ml; Sigma) in a shaking incubator at 37°C for ∼60 min according to a modification of the Rodbell method [47]. The digested tissue was filtered and the isolated cells were washed twice in Krebs-Ringer buffer (pH 7.4) with 1% BSA, 2 mM glucose and 200 nM adenosine by allowing the isolated adipocytes to float to the surface and then aspirate the underlying buffer.

### Palmitate oxidation in isolated white adipocytes

Palmitate oxidation in isolated adipocytes of perigonadal WAT from 6 months old wildtype and HSL null mice fed a normal chow diet was determined by production of ^14^CO_2_ from [1-^14^C]-palmitate as described [48]. Briefly, adipocytes were isolated as above, washed thoroughly and transferred into an assay buffer containing 114 mM NaCl, 4.7 mM KCl, 1.2 mM KH_2_PO_4_, 1.16 mM MgSO4, 25 mM NaHCO_3_, 2.5 mM CaCl_2_, 20 mM HEPES pH 7.2 and 3 mM glucose. Cell density was estimated after which cell suspensions (10%) were allowed to recover at 37°C for 30 min prior to the oxidation assay. Hundred µl of cell suspension was transferred to a sample cup together with a solution, containing 5 mM palmitate (unlabeled and 0.5 µCi [1-^14^C] palmitate) (Du-Pont, NEN, specific activity 55.8 mCi/mmol) and 16.7 mM glucose. The sample cups were sealed with rubber stoppers into glass vials, and incubated at 37°C for one hour. The reaction was terminated by injection of 100 µl of 7% trichloroacetic acid (TCA) into the sample cup. The released ^14^CO_2_ was captured by adding 300 µl of benzethonium hydroxide into the glass vial and the radioactivity was determined in a liquid scintillation counter.

### Statistics

Data are expressed as mean±SEM unless otherwise stated. Statistical analyses were made using either a nonparametric Mann-Whitney U test or by one-way analysis of variance followed by a Bonferroni post hoc test. Data were considered significant if P<0.05. P<0.05 = *, P<0.01 = **, P<0.001 = ***.

## References

[1] Umek RM, Friedman AD, McKnight SL (1991). CCAAT-enhancer binding protein: a component of a differentiation switch.. Science.

[2] Rosen ED, Sarraf P, Troy AE, Bradwin G, Moore K (1999). PPAR gamma is required for the differentiation of adipose tissue in vivo and in vitro.. Mol Cell.

[3] Tontonoz P, Hu E, Spiegelman BM (1994). Stimulation of adipogenesis in fibroblasts by PPAR gamma 2, a lipid-activated transcription factor.. Cell.

[4] Picard F, Gehin M, Annicotte J, Rocchi S, Champy MF (2002). SRC-1 and TIF2 control energy balance between white and brown adipose tissues.. Cell.

[5] Hansen JB, Jorgensen C, Petersen RK, Hallenborg P, De Matteis R (2004). Retinoblastoma protein functions as a molecular switch determining white versus brown adipocyte differentiation.. Proc Natl Acad Sci U S A.

[6] Puigserver P, Wu Z, Park CW, Graves R, Wright M (1998). A cold-inducible coactivator of nuclear receptors linked to adaptive thermogenesis.. Cell.

[7] Uldry M, Yang W, St-Pierre J, Lin J, Seale P (2006). Complementary action of the PGC-1 coactivators in mitochondrial biogenesis and brown fat differentiation.. Cell Metab.

[8] White R, Morganstein D, Christian M, Seth A, Herzog B (2008). Role of RIP140 in metabolic tissues: Connections to disease.. FEBS Lett.

[9] Leonardsson G, Steel JH, Christian M, Pocock V, Milligan S (2004). Nuclear receptor corepressor RIP140 regulates fat accumulation.. Proc Natl Acad Sci U S A.

[10] Timmons JA, Wennmalm K, Larsson O, Walden TB, Lassmann T (2007). Myogenic gene expression signature establishes that brown and white adipocytes originate from distinct cell lineages.. Proc Natl Acad Sci U S A.

[11] Yeaman SJ (2004). Hormone-sensitive lipase–new roles for an old enzyme.. Biochem J.

[12] Fredrikson G, Stralfors P, Nilsson NO, Belfrage P (1981). Hormone-sensitive lipase of rat adipose tissue. Purification and some properties.. J Biol Chem.

[13] Wei S, Lai K, Patel S, Piantedosi R, Shen H (1997). Retinyl ester hydrolysis and retinol efflux from BFC-1beta adipocytes.. J Biol Chem.

[14] Osuga J, Ishibashi S, Oka T, Yagyu H, Tozawa R (2000). Targeted disruption of hormone-sensitive lipase results in male sterility and adipocyte hypertrophy, but not in obesity.. Proc Natl Acad Sci U S A.

[15] Wang SP, Laurin N, Himms-Hagen J, Rudnicki MA, Levy E (2001). The adipose tissue phenotype of hormone-sensitive lipase deficiency in mice.. Obes Res.

[16] Haemmerle G, Zimmermann R, Hayn M, Theussl C, Waeg G (2002). Hormone-sensitive lipase deficiency in mice causes diglyceride accumulation in adipose tissue, muscle, and testis.. J Biol Chem.

[17] Mulder H, Sorhede-Winzell M, Contreras JA, Fex M, Strom K (2003). Hormone-sensitive lipase null mice exhibit signs of impaired insulin sensitivity whereas insulin secretion is intact.. J Biol Chem.

[18] Harada K, Shen WJ, Patel S, Natu V, Wang J (2003). Resistance to high-fat diet-induced obesity and altered expression of adipose-specific genes in HSL-deficient mice.. Am J Physiol Endocrinol Metab.

[19] Sekiya M, Osuga J, Okazaki H, Yahagi N, Harada K (2004). Absence of hormone-sensitive lipase inhibits obesity and adipogenesis in Lep ob/ob mice.. J Biol Chem.

[20] Fex M, Olofsson CS, Fransson U, Bacos K, Lindvall H (2004). Hormone-sensitive lipase deficiency in mouse islets abolishes neutral cholesterol ester hydrolase activity but leaves lipolysis, acylglycerides, fat oxidation, and insulin secretion intact.. Endocrinology.

[21] Hansson O, Donsmark M, Ling C, Nevsten P, Danfelter M (2005). Transcriptome and proteome analysis of soleus muscle of hormone-sensitive lipase-null mice.. J Lipid Res.

[22] Voshol PJ, Haemmerle G, Ouwens DM, Zimmermann R, Zechner R (2003). Increased hepatic insulin sensitivity together with decreased hepatic triglyceride stores in hormone-sensitive lipase-deficient mice.. Endocrinology.

[23] Park SY, Kim HJ, Wang S, Higashimori T, Dong J (2005). Hormone-sensitive lipase knockout mice have increased hepatic insulin sensitivity and are protected from short-term diet-induced insulin resistance in skeletal muscle and heart.. Am J Physiol Endocrinol Metab.

[24] Roduit R, Masiello P, Wang SP, Li H, Mitchell GA (2001). A role for hormone-sensitive lipase in glucose-stimulated insulin secretion: a study in hormone-sensitive lipase-deficient mice.. Diabetes.

[25] Cinti S, Mitchell G, Barbatelli G, Murano I, Ceresi E (2005). Adipocyte death defines macrophage localization and function in adipose tissue of obese mice and humans.. J Lipid Res.

[26] Hansson O, Strom K, Guner N, Wierup N, Sundler F (2006). Inflammatory response in white adipose tissue in the non-obese hormone-sensitive lipase null mouse model.. J Proteome Res.

[27] Zimmermann R, Haemmerle G, Wagner EM, Strauss JG, Kratky D (2003). Decreased fatty acid esterification compensates for the reduced lipolytic activity in hormone-sensitive lipase-deficient white adipose tissue.. J Lipid Res.

[28] Ntambi JM, Buhrow SA, Kaestner KH, Christy RJ, Sibley E (1988). Differentiation-induced gene expression in 3T3-L1 preadipocytes. Characterization of a differentially expressed gene encoding stearoyl-CoA desaturase.. J Biol Chem.

[29] Brown NF, Hill JK, Esser V, Kirkland JL, Corkey BE (1997). Mouse white adipocytes and 3T3-L1 cells display an anomalous pattern of carnitine palmitoyltransferase (CPT) I isoform expression during differentiation. Inter-tissue and inter-species expression of CPT I and CPT II enzymes.. Biochem J.

[30] Fortier M, Wang SP, Mauriege P, Semache M, Mfuma L (2004). Hormone-sensitive lipase-independent adipocyte lipolysis during beta-adrenergic stimulation, fasting, and dietary fat loading.. Am J Physiol Endocrinol Metab.

[31] Glass CK, Rose DW, Rosenfeld MG (1997). Nuclear receptor coactivators.. Curr Opin Cell Biol.

[32] Hansen JB, Kristiansen K (2006). Regulatory circuits controlling white versus brown adipocyte differentiation.. Biochem J.

[33] Christian M, White R, Parker MG (2006). Metabolic regulation by the nuclear receptor corepressor RIP140.. Trends Endocrinol Metab.

[34] Schweiger M, Schreiber R, Haemmerle G, Lass A, Fledelius C (2006). Adipose triglyceride lipase and hormone-sensitive lipase are the major enzymes in adipose tissue triacylglycerol catabolism.. J Biol Chem.

[35] Zimmermann R, Strauss JG, Haemmerle G, Schoiswohl G, Birner-Gruenberger R (2004). Fat mobilization in adipose tissue is promoted by adipose triglyceride lipase.. Science.

[36] Tsukiyama-Kohara K, Poulin F, Kohara M, DeMaria CT, Cheng A (2001). Adipose tissue reduction in mice lacking the translational inhibitor 4E-BP1.. Nat Med.

[37] Soloveva V, Graves RA, Rasenick MM, Spiegelman BM, Ross SR (1997). Transgenic mice overexpressing the beta 1-adrenergic receptor in adipose tissue are resistant to obesity.. Mol Endocrinol.

[38] Cederberg A, Gronning LM, Ahren B, Tasken K, Carlsson P (2001). FOXC2 is a winged helix gene that counteracts obesity, hypertriglyceridemia, and diet-induced insulin resistance.. Cell.

[39] Christian M, Kiskinis E, Debevec D, Leonardsson G, White R (2005). RIP140-targeted repression of gene expression in adipocytes.. Mol Cell Biol.

[40] Kerley JS, Olsen SL, Freemantle SJ, Spinella MJ (2001). Transcriptional activation of the nuclear receptor corepressor RIP140 by retinoic acid: a potential negative-feedback regulatory mechanism.. Biochem Biophys Res Commun.

[41] Ribot J, Oliver P, Serra F, Palou A (2005). Retinoic acid modulates the retinoblastoma protein during adipocyte terminal differentiation.. Biochim Biophys Acta.

[42] Grober J, Lucas S, Sorhede-Winzell M, Zaghini I, Mairal A (2003). Hormone-sensitive lipase is a cholesterol esterase of the intestinal mucosa.. J Biol Chem.

[43] Schwarz M, Lund EG, Setchell KD, Kayden HJ, Zerwekh JE (1996). Disruption of cholesterol 7alpha-hydroxylase gene in mice. II. Bile acid deficiency is overcome by induction of oxysterol 7alpha-hydroxylase.. J Biol Chem.

[44] Pecqueur C, Alves-Guerra MC, Gelly C, Levi-Meyrueis C, Couplan E (2001). Uncoupling protein 2, in vivo distribution, induction upon oxidative stress, and evidence for translational regulation.. J Biol Chem.

[45] Matthias A, Ohlson KB, Fredriksson JM, Jacobsson A, Nedergaard J (2000). Thermogenic responses in brown fat cells are fully UCP1-dependent. UCP2 or UCP3 do not substitute for UCP1 in adrenergically or fatty scid-induced thermogenesis.. J Biol Chem.

[46] Cannon B, Nedergaard J, Ailhaud G (2001). Respiratory and Thermogenic Capacities of Cells and Mitochondria from Brown and White Adipose Tissue.. Methods in Molecular Biology: Adipose Tissue Protocols.

[47] Rodbell M (1964). Metabolism of Isolated Fat Cells. I. Effects of Hormones on Glucose Metabolism and Lipolysis.. J Biol Chem.

[48] Antinozzi PA, Segall L, Prentki M, McGarry JD, Newgard CB (1998). Molecular or pharmacologic perturbation of the link between glucose and lipid metabolism is without effect on glucose-stimulated insulin secretion. A re-evaluation of the long-chain acyl-CoA hypothesis.. J Biol Chem.

